# The miracle of IVUS for unforeseen stent thrombosis: A case report

**DOI:** 10.1002/ccr3.8935

**Published:** 2024-05-24

**Authors:** Maryam Mehrpooya, Parisa Koohsari, Ehsan Moradi Farsani

**Affiliations:** ^1^ Department of Cardiology Imam Khomeini Hospital Complex Tehran University of Medical Sciences Tehran Iran; ^2^ School of Medicine Imam Khomeini Hospital Complex Tehran University of Medical Sciences Tehran Iran

**Keywords:** intravascular ultrasonography, malapposition, postdilation, stent thrombosis, thrombosuction, under‐expansion

## Abstract

Stent thrombosis is a serious complication with high morbidity and mortality rates resulting in cardiac death or nonfatal myocardial infarction that occurs following stent placement during percutaneous coronary intervention (PCI). Stent underexpansion or malapposition are avoidable risk factors for stent thrombosis. Sufficient postdilation should be considered to mitigate this risk, especially with the guidance of intravascular ultrasound (IVUS). We present the case of a 60‐year‐old man developing a thrombotic lesion inside a stent 2 weeks after PCI for Non‐ST‐Segment Elevation Myocardial Infarction (NSTEMI), which was strongly related to stent underexpansion and malapposition. This case highlights the importance of IVUS in evaluating procedural success, particularly in assessing stent expansion and apposition.

## 1 INTRODUCTION

Stent thrombosis (ST) is a complication of percutaneous coronary intervention (PCI) with catastrophic clinical consequences and an incidence rate of 3%–20%.^1^ Stent underexpansion and malapposition are the major causes of this complication that can be well‐diagnosed and prevented by intracoronary imaging like intravascular ultrasound (IVUS) and optical coherence tomography (OCT).^2^

This report presents a 60‐year‐old gentleman with a history of recent PCI on RCA (due to inferior NSTEMI). RCA stent thrombosis (due to underexpansion and malapposition) was diagnosed during staged PCI for LCX‐OM which was successfully managed under the guidance of IVUS.

## 2 CASE HISTORY

The patient, a 60‐year‐old non‐smoker man with a history of hypertension, dyslipidemia, and PCI on the left anterior descending (LAD) artery 3 years ago was admitted to our hospital with severe chest pain, dyspnea, and diaphoresis. In electrocardiography, sinus rhythm and ST depression (4 mm) were seen in inferior leads (II, III, and aVF). Troponin I level was elevated to 20 times of normal reference value.

## 3 METHODS

**FIGURE 1:**
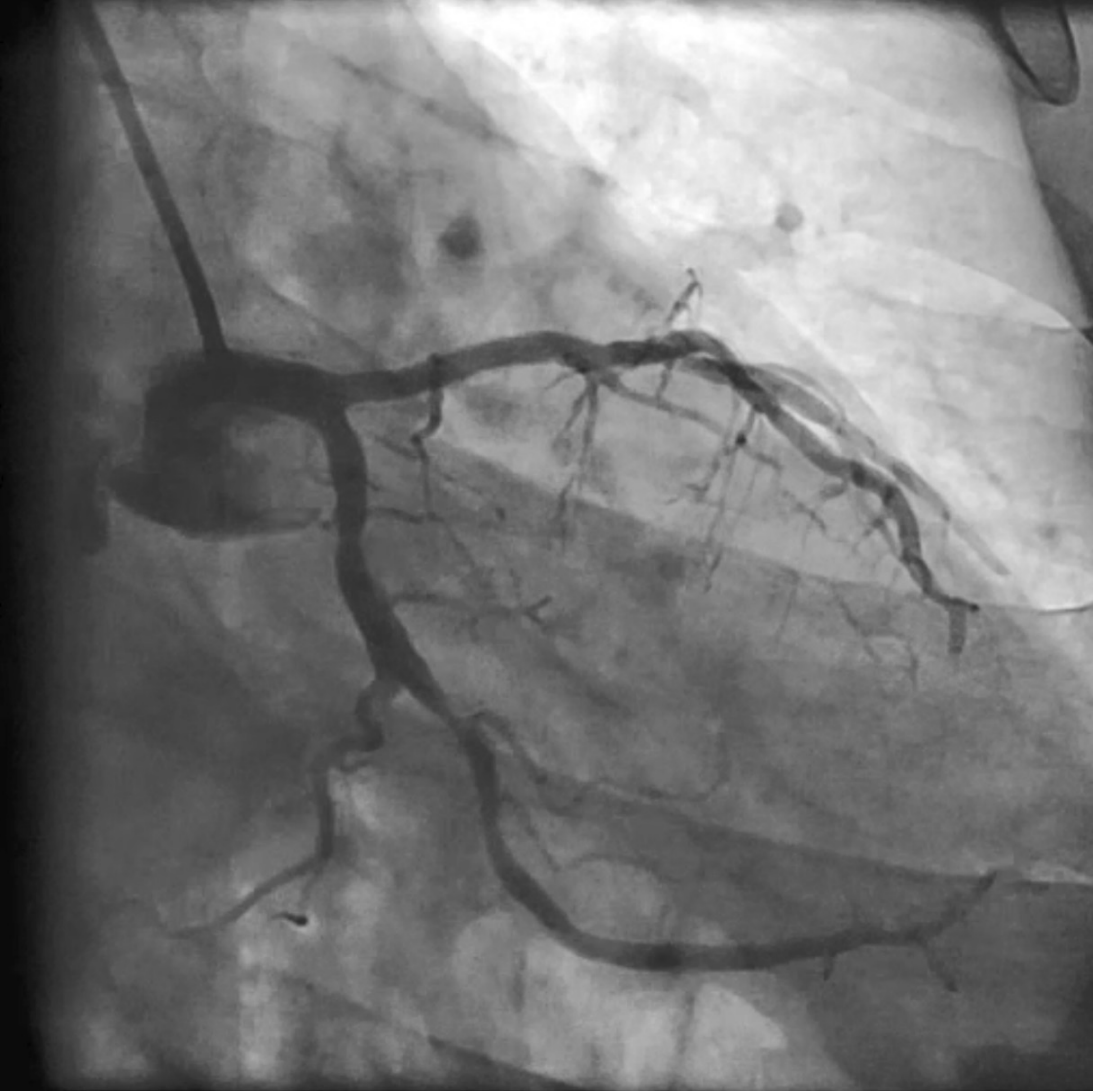
Significant lesions in the LCX and OM arteries.

**FIGURE 2:**
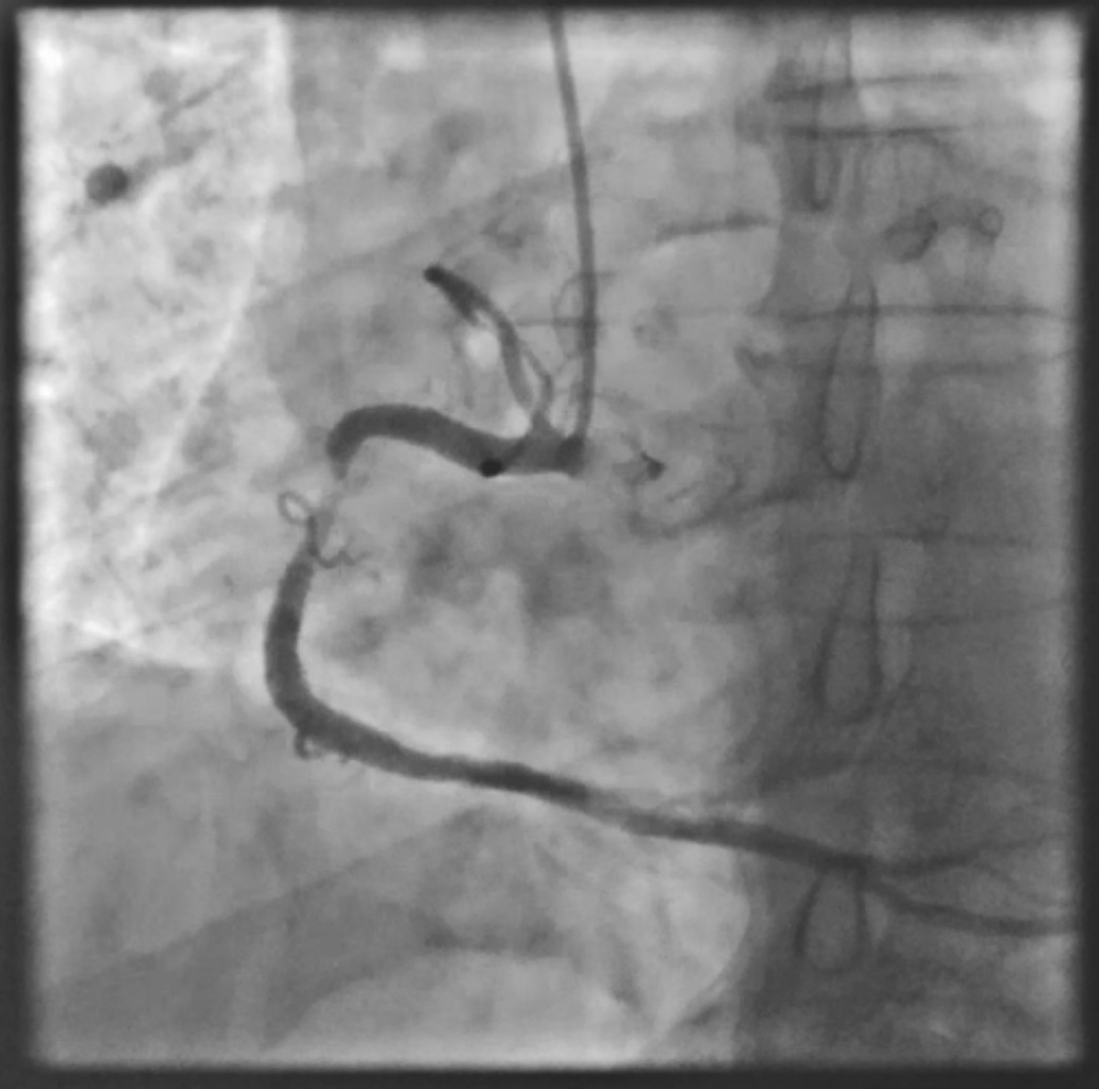
Thrombotic lesion in the proximal to mid part of the RCA.

**FIGURE 3:**
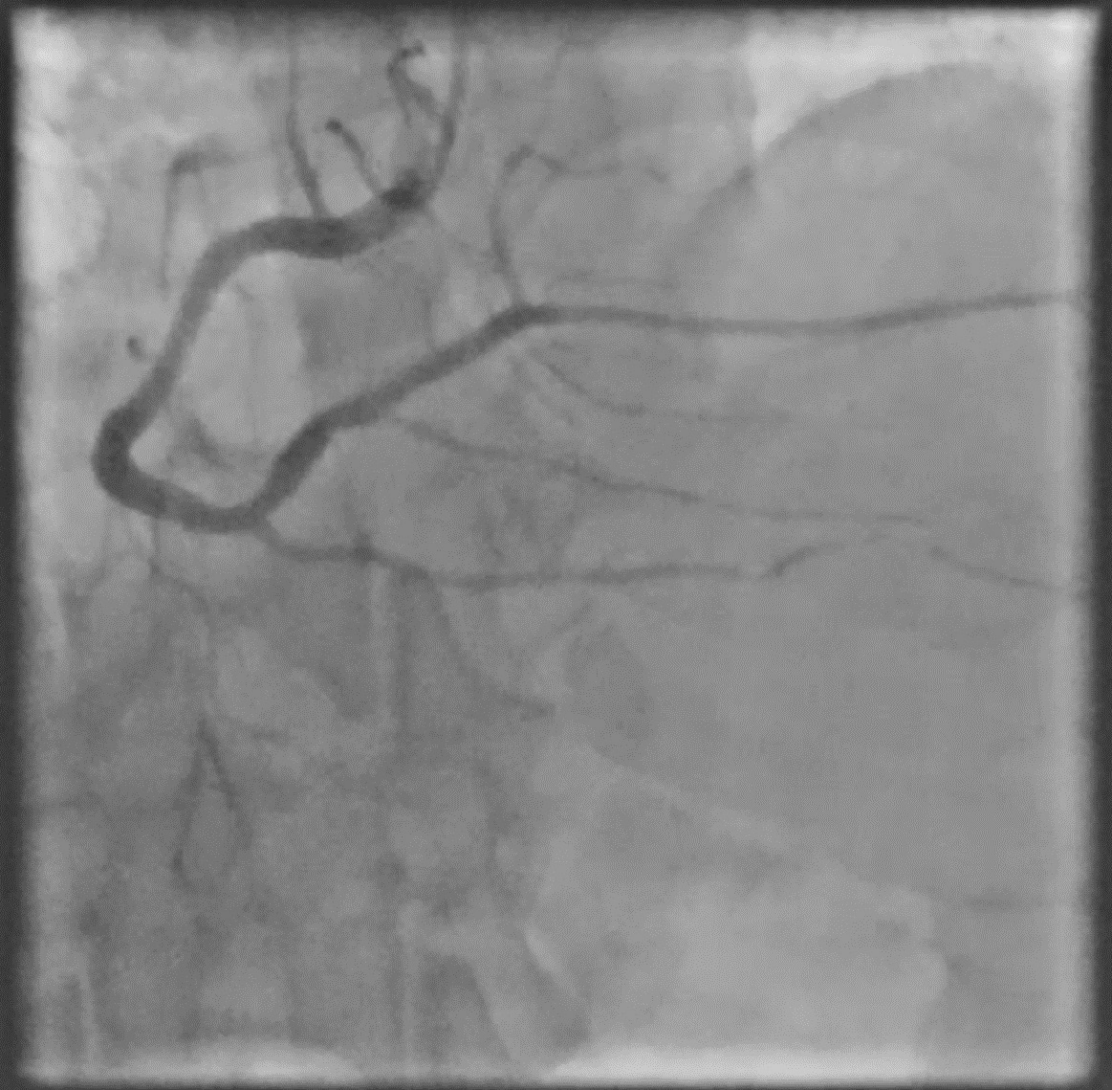
PCI on RCA (first session).

He was diagnosed with non‐ST‐segment elevation myocardial infarction (NSTEMI). He underwent coronary angiography via the right radial artery, which revealed significant lesions in the LCX and OM arteries, moderate (50%–60%) stenosis in the distal edge of the LAD artery stent (Figure 1, Movie 1), as well as a thrombotic lesion in the proximal to mid part of the RCA (Figure 2, Movie 2). PCI was performed on the RCA using Xience Alpine 3.5*33 mm stent, followed by postdilation that seemed to be enough by NC Saphaire and Vecchio 3.5*18 mm balloons (Figure 3, Movie 3).

**Movie 1:** Significant lesions in the LCX and OM arteries.

**Movie 2:** Thrombotic lesion in the proximal to mid part of the RCA.

**Movie 3:** PCI on RCA (first session).

Staged PCI for LCX‐OM was scheduled 2 weeks later regarding borderline renal function (Cr = 1.3 mg/dL) and after hydration and renal support. Notably, the patient complied well with treatment and received aspirin and clopidogrel as standard strategy. Due to the patient's previous hypersensitivity to ticagrelor resulting in pruritus during a PCI procedure 3 years ago, clopidogrel was chosen for this current procedure. PCI was successfully performed for LCX by Xience Alpine 3.5*15 mm stent and OM by Supraflex 2.75*28 mm stent.

**FIGURE 4:**
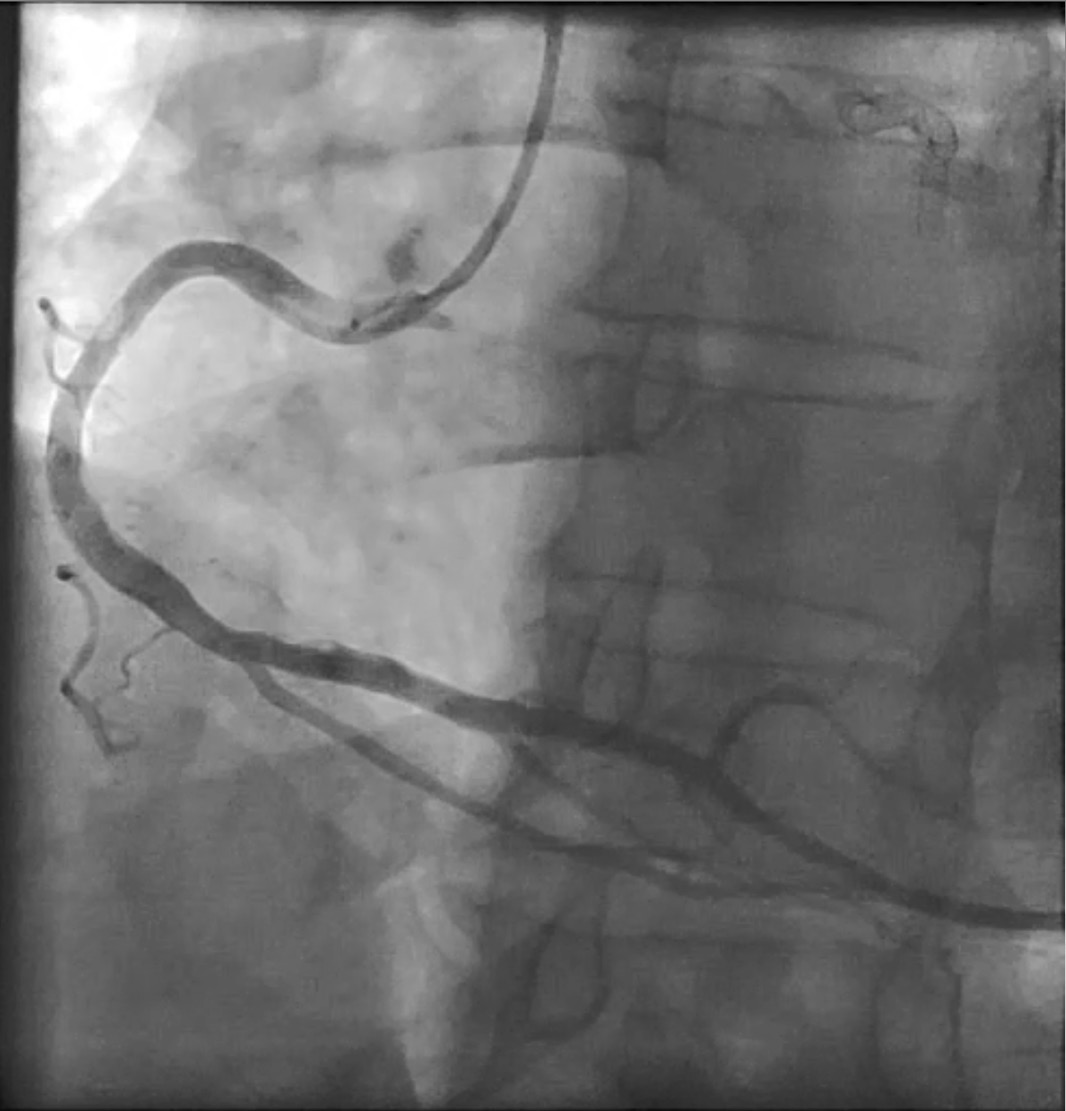
Thrombotic lesion in the middle of the RCA stent (next session).

**FIGURE 5:**
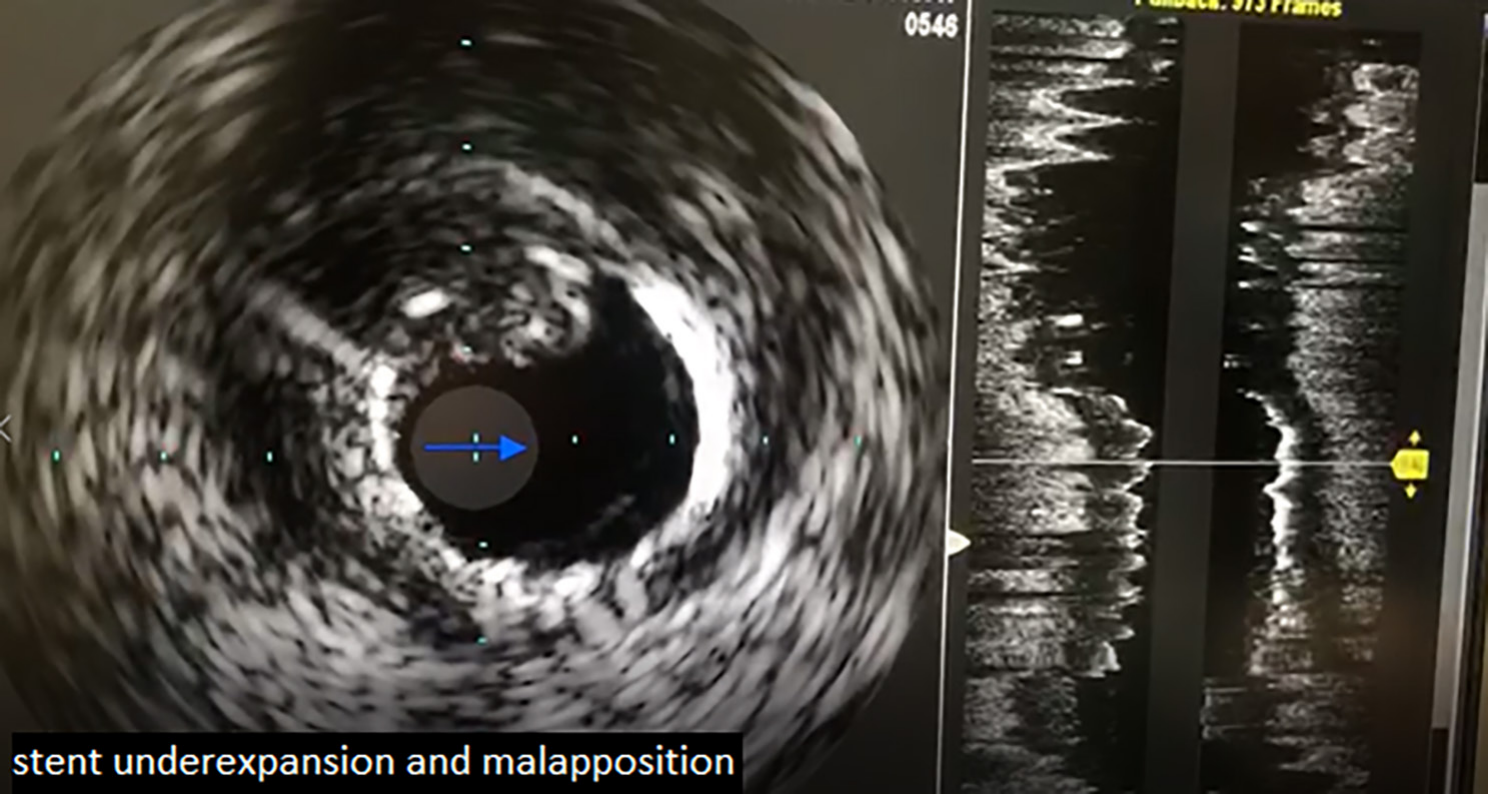
Semi‐fresh thrombus in the middle of stent with malapposition & underexpansion in IVUS.

The previously implanted stent in RCA was evaluated as well. Unexpectedly, a thrombotic lesion located within the middle segment of the stent was encountered (Figure 4 and Movie 4). According to the observed problem, we decided to perform IVUS for further assistance. The IVUS examination revealed the presence of a semi‐fresh thrombus within the middle portion of the RCA stent, accompanied by remarkable stent underexpansion also malapposition, as depicted in Figure 5 and Movie 5. Therefore, we performed thrombosuction by Capturer thrombus extraction catheter and postdilation by NC TREK 4*15 mm balloon.

**Movie 4:** Thrombotic lesion in the middle of the RCA stent (next session).

**Movie 5:** Semi‐fresh thrombus in the middle of stent with malapposition and underexpansion in IVUS.

## 4 CONCLUSION AND RESULTS

**FIGURE 6:**
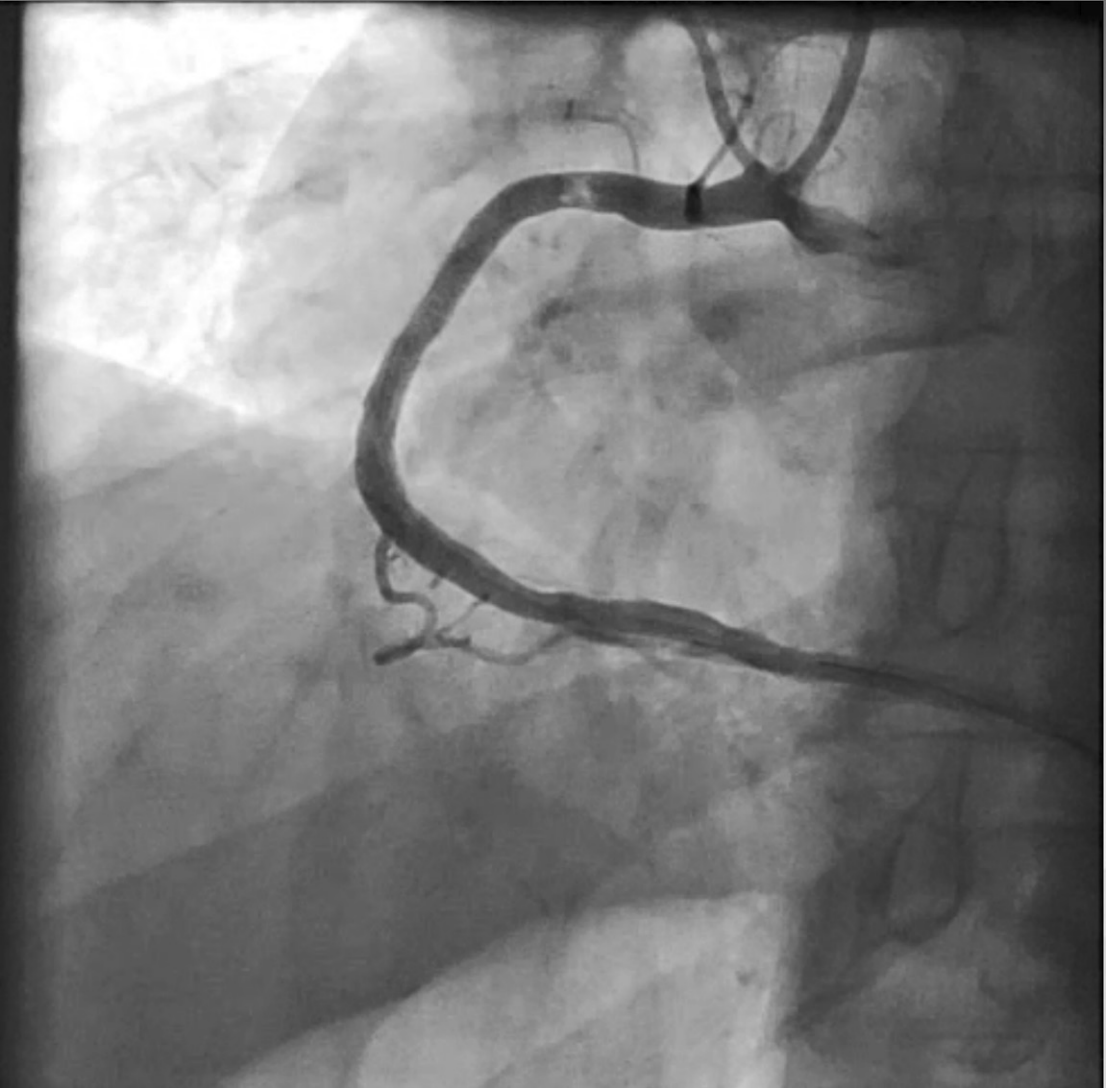
Mobile clot in proximal of RCA.

**FIGURE 7:**
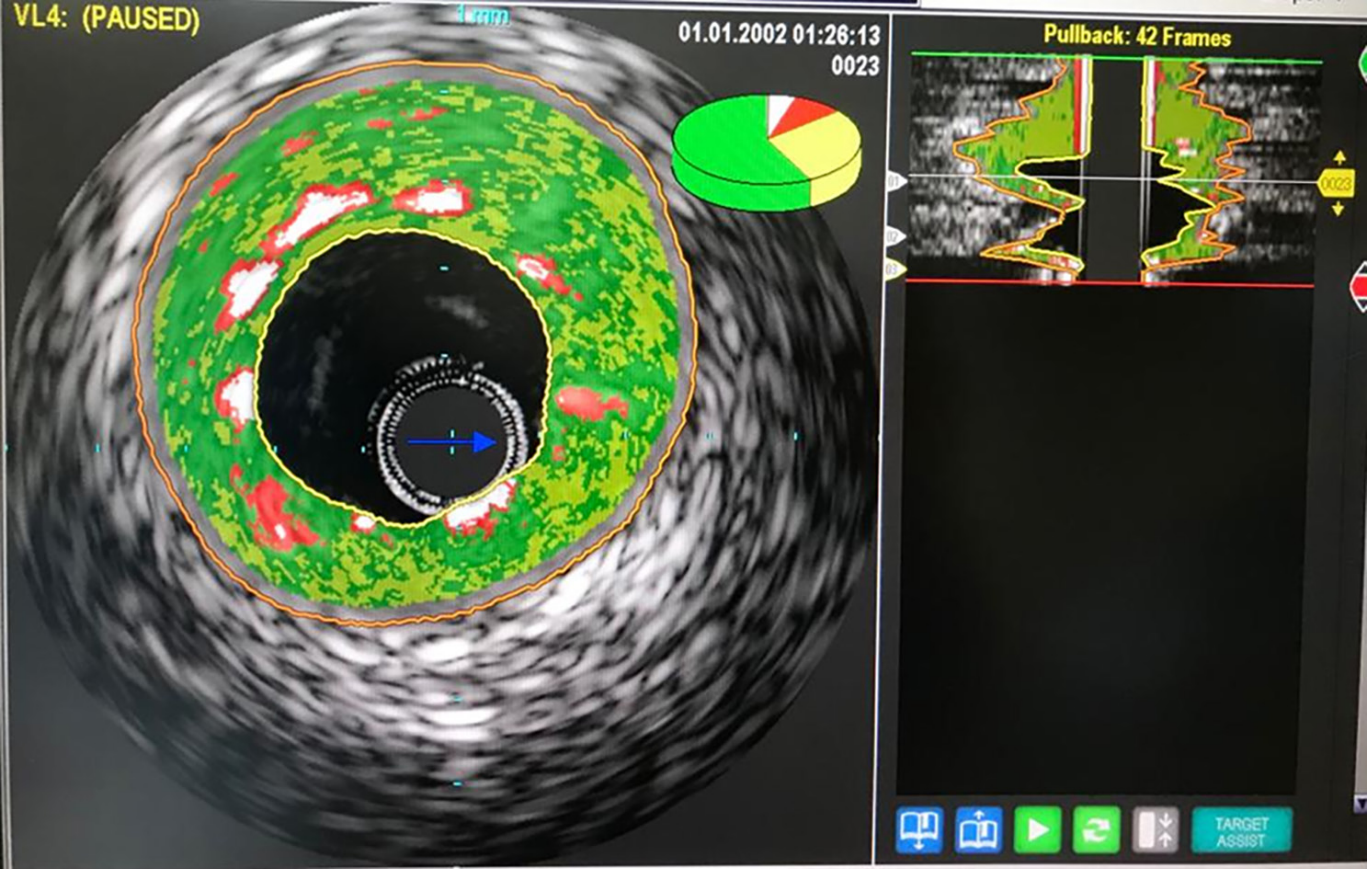
Complete expansion and apposition of the stent struts in IVUS.

During the final control injection, a mobile clot was detected in the proximal RCA (Figure 6, Movie 6), necessitating another round of thrombosuction. However, subsequent angiography and IVUS study revealed TIMI grade 3 flow in the RCA and demonstrated complete expansion and apposition of the stent struts (Figure 7, Movie 7). As a result, the patient was discharged 2 days later in a stable condition. This case highlights the importance of IVUS in evaluating procedural success, especially for assessing stent expansion and apposition.

**Movie 6:** Mobile clot in proximal of RCA.

**Movie 7:** Complete expansion and apposition of the stent struts in angiography.

## 5 DISCUSSION

ST is a rare but devastating complication of PCI with up to 50% mortality rate in early cases.^3^ The multifactorial causes of ST include factors related to the patient, the procedure, and thrombogenicity. Acute coronary syndrome (ACS), reduced kidney function (considering GFR), history of previous coronary artery disease (CAD), uncontrolled hypertension, and hyperlipidemia were among the possible patient‐related factors causing stent thrombosis in our case. However, a meta‐analysis on factors impacting ST questioned the clinical significance of baseline characteristics in terms of predicting ST in PCI patients due to high heterogeneity in outcomes.^4^

Stent underexpansion, stent under‐sizing, geographic miss, edge dissection, in‐stent tissue protrusion/prolapse, acute stent malapposition, stent fracture, longitudinal stent deformation, and non‐uniform strut distribution are stent‐related problems which can cause ST and can be detected by intravascular imaging like IVUS and OCT.^2^ We detected stent underexpansion in IVUS imaging of our patient, despite the sufficient postdilation performed 2 weeks ago after stent implantation.

IVUS is an imaging modality used to characterize lesion morphology, quantify plaque burden, guide stent sizing, assess stent expansion, stent apposition and identify procedural complications by obtaining a 360‐degree view of the vessel.^5^ In our case, IVUS played a key role in the diagnosis of ST. The stent thrombosis appeared to be attributed to procedure‐related factors, specifically stent underexpansion, and malapposition after first PCI.

Randomized trials have demonstrated that an IVUS‐guided revascularization strategy compared with angiography‐guided PCI can lead to improved clinical outcomes and is associated with a reduction in major adverse cardiovascular events (MACE).^6^ This was explained with more postdilation and larger stent sizes, final larger angiographic minimal lumen diameters, and larger minimal stent areas while using IVUS, which minimized stent underexpansion. Also, more stents were implanted and longer stents were used with IVUS guidance to minimize geographic miss and treat edge dissections.^6^

Stent underexpansion is a major risk factor of stent thrombosis, defined as when the stent doesn't reach nominal stent size but stent struts reach the vessel wall. Post‐PCI inadequate minimal stent area (MSA) is consistently the strongest predictor of stent thrombosis.^2^

A systematic review and meta‐analysis comparing IVUS‐guided vs. angiography‐guided chronic total occlusions (CTO) PCI (*n* = 2320 patients) revealed that IVUS guidance was associated with significantly longer stents and larger diameters (*p* = 0.007, *p* < 0.001, respectively). Also, stent thrombosis was the only secondary clinical outcome that showed a significant difference, favoring the IVUS‐guided approach (*p* = 0.01), while the primary outcome of MACE did not significantly differ between the groups (*p* = 0.40). These results underscore the potential benefits of routine IVUS guidance in CTO‐PCI procedures to mitigate the catastrophic implications of stent thrombosis.^7^

Furthermore, histopathological, intravascular ultrasound (IVUS), and optical coherence tomography (OCT) analyses have indicated a high incidence of secondary plaque ruptures in patients presenting with acute coronary syndrome (ACS), estimated at approximately 25%. Consequently, observational clinical studies have demonstrated potential benefits in reducing the rates of cardiovascular mortality, recurrent infarction, and ischemia‐driven revascularization through the routine performance of percutaneous coronary intervention (PCI) on non‐culprit arteries in non‐ST‐elevation myocardial infarction (NSTEMI) cases. These findings underscore the critical role of IVUS in guiding such interventions.^8^

ST is a medical emergency that may be associated with death, myocardial infarction, and significant cardiac morbidity, which mainly requires revascularization. This case emphasized IVUS as a highly useful modality for investigating the causal mechanisms of ST, as it reveals pathophysiologic factors underestimated by conventional angiography and identifies patients who may benefit from more additional coronary interventions, and it should be at the center of our attention.

## AUTHOR CONTRIBUTIONS

**Maryam Mehrpooya:** Project administration; supervision; visualization; writing – original draft; writing – review and editing. **Parisa Koohsari:** Visualization; writing – original draft; writing – review and editing. **Ehsan Moradi Farsani:** Visualization; writing – original draft; writing – review and editing.

## FUNDING INFORMATION

None.

## CONFLICT OF INTEREST STATEMENT

The authors declare no conflicts of interest.

## ETHICS STATEMENT

The study was approved by the Medical Ethics Committee of Tehran University of Medical Sciences.

## CONSENT

Written informed consent was obtained from the patient to publish this report in accordance with the journal's patient consent policy.

## PERMISSION TO REPRODUCE MATERIAL FROM OTHER SOURCES

N/A.

## Data Availability

Data are available on request from the authors.
